# Single Nucleotide Variant rs2232710 in the Protein Z-Dependent Protease Inhibitor (*ZPI*, *SERPINA10*) Gene Is Not Associated with Deep Vein Thrombosis

**DOI:** 10.1371/journal.pone.0151347

**Published:** 2016-03-16

**Authors:** Marcin M. Gorski, Luca A. Lotta, Emanuela Pappalardo, Hugoline G. de Haan, Serena M. Passamonti, Astrid van Hylckama Vlieg, Ida Martinelli, Flora Peyvandi

**Affiliations:** 1 Department of Pathophysiology and Transplantation, Università degli Studi di Milano, Milan, Italy; 2 Angelo Bianchi Bonomi Hemophilia and Thrombosis Center, Fondazione IRCCS Cà Granda - Ospedale Maggiore Policlinico and Fondazione Luigi Villa, Milan, Italy; 3 Departments of Clinical Epidemiology, Leiden University Medical Center, Leiden, Netherlands; Medical University Innsbruck, AUSTRIA

## Abstract

Rare mutations in *PROC*, *PROS1* or *SERPINC1* as well as common variants in *F5*, *F2*, *F11* and *SERPINC1* have been identified as risk factors for deep vein thrombosis (DVT). To identify novel genetic risk factors for DVT, we have developed and applied next-generation DNA sequencing (NGS) of the coding area of hemostatic and proinflammatory genes. Using this strategy, we previously identified a single nucleotide variant (SNV) rs6050 in the *FGA* gene and novel, rare SNVs in the *ADAMTS13* gene associated with DVT. To identify novel coding variants in the genetic predisposition to DVT, we applied NGS analysis of the coding area of 186 hemostatic and proinflammatory genes in 94 DVT cases and 98 controls and we identified 18 variants with putative role in DVT. A group of 585 Italian idiopathic DVT patients and 550 healthy controls was used to genotype all the 18 risk-associated variants identified by NGS. Replication study in the Italian population identified the rs2232710 variant in the protein Z-dependent protease inhibitor (*ZPI*) gene to be associated with an increased risk of DVT (OR 2.74; 95% CI 1.33–5.65; *P* = 0.0045; Bonferroni *P* = 0.081). However, the rs2232710 SNV showed no association with DVT in two Dutch replication cohorts the LETS study (454 patients and 451 controls) and the MEGA study (3799 patients and 4399 controls), indicating that the rs2232710 variant is not a risk factor for DVT.

## Introduction

Deep vein thrombosis (DVT) of the lower extremities has a strong genetic basis, with an estimated hereditary component of 60%, but established genetic risk factors for DVT explain only a fraction of disease heritability [1,2]. Genetic risk factors include rare mutations in genes encoding natural anticoagulant proteins such as antithrombin, protein C, protein S or single nucleotide polymorphisms (SNPs) in the *F5* gene (rs6025 or Factor V Leiden [FVL]) that impair down-regulation of procoagulant pathway or in the *F2* gene (rs1799963 or G20120A) that result in increased levels of prothrombin [3,4]. Moreover, polymorphisms in several genomic loci such as *F11*, *SERPINC1*, *HIVEP1*, *GP6*, *TSPAN15* and *SLC44A2* have recently been identified in genome-wide association screens (GWAS) as susceptibility loci for DVT [5–8].

The role of the protein Z (PZ) and protein Z-dependent inhibitor (ZPI) pathway in venous thromboembolism has been recently assessed in clinical studies and using murine models [9]. ZPI is a single-chain glycoprotein that together with its vitamin K-dependent glycoprotein cofactor PZ inhibits activated coagulation factors X and XI [9]. Several non-synonymous variants in the *ZPI*/*SERPINA10* gene (henceforth called *ZPI*), in particular R67X and W303X mutations, have been identified in patients suffering from thrombophilia [10], even though other studies did not support these findings [11–13]. Similarly, the association of the PZ-ZPI pathway with arterial diseases, ischemic stroke, or unexplained early pregnancy loss yielded conflicting results [14–18]. In animal model, the knockout (KO) of either *ZP* or *ZPI* gene resulted in enhanced thrombotic phenotype following arterial injury [19]. However, the combination of ZPI deficiency with the homozygous FVL variant led to a more severe thrombotic phenotype than *PZ* KO/FVL, implying an important role for the ZPI protein in the inhibition of activated XI [19]. Recently, two missense mutations F145L and Q384R in the *ZPI* gene were shown to impair the inhibitory activity of the ZPI protein *in vitro*, even though they were not associated with DVT in humans [20].

The advent of powerful ‘next-generation’ DNA sequencing technology offers unprecedented opportunities to identify genetic risk factors for complex disorders such as deep vein thrombosis. These novel technologies enable sequencing of large proportions of the human genome (or even the entire genome) at unparalleled speed and costs. Deep sequencing of all protein-coding areas of the genome is rapidly becoming the gold standard for the identification of causal genes underlying the development of both common and rare Mendelian diseases [21–24]. To assess the potential role of rare coding variants underlying genetic predisposition to venous thrombosis, we have recently developed and successfully applied targeted next-generation DNA sequencing-based (NGS) strategy and analysis of the coding area of 186 hemostatic and proinflammatory genes in Italian idiopathic DVT cases and controls. Using this approach, we identified a non-synonymous variant rs6050 in the *FGA* gene [25] as well as several rare coding single nucleotide variants (SNVs) in the *ADAMTS13* gene [26] as risk factors for DVT. The aim of this study was to assess the potential role of non-synonymous coding variants explaining genetic predisposition to DVT.

## Materials and Methods

### Participants

The details of the recruitment of DVT patients and healthy controls have been described elsewhere [25]. Briefly, DVT patients and healthy controls for this case-control study were from the DVT-Milan study. A total of 2139 unrelated Italian patients with DVT and 1938 healthy controls were recruited to the Angelo Bianchi Bonomi Hemophilia and Thrombosis Center (Milan, Italy) between 1995 and 2010. For this study, we identified 719 unrelated idiopathic DVT cases that were diagnosed for DVT of the lower limbs. DVT cases were selected according to the following criteria: (i) objective diagnosis of DVT; (ii) Caucasian ethnicity born from Caucasian parent; (iii) absence of cancer or surgery associated with DVT; (iv) absence of natural anticoagulant deficiencies determined by natural levels of protein C, protein S and antithrombin in routine testing; (v) absence of factor V Leiden and prothrombin G20120A variants determined by sequencing; and (vi) signed informed consent. The study was approved by the Medical Ethics Committee of the Fondazione IRCCS Ca’ Granda, Hospital Maggiore and has been carried out in accordance with the code of ethics of the World Medical Association (Declaration of Helsinki). Patients recruitment, sampling and thrombophilia screening was performed at the Angelo Bianchi Bonomi Hemophilia and Thrombosis Center in Milan, Italy. The next-generation DNA sequencing was performed on 94 idiopathic DVT cases and 98 controls (discovery phase) at the Human Genome Sequencing Center, Baylor College of Medicine, Houston, TX, USA. Replication in the Italian patients and controls as well as in the two Dutch case-control studies were carried out at the Leiden University Medical Center, Leiden, Netherlands.

### Sequencing and data analysis (discovery phase)

Data presented in this article have been sequenced and analyzed as a part of work previously described by Lotta et al. [25,26]. The protein-coding regions and intron-exon boundaries of 186 candidate hemostatic and proinflammatory genes were sequenced in 94 Italian cases of idiopathic DVT and 98 healthy controls using Applied Biosystems SOLiD 4 sequencing system at the Human Genome Sequencing Centre (HGSC) at Baylor College of Medicine, Houston, USA. A gene list is provided elsewhere [25,26]. Variant calling steps included data analysis on raw reads to produce individual binary alignment/mapping (BAM) files and PILEUP files using SAMtools [27]. Variants not meeting quality control criteria as specified by the HGSC were removed. Genetic variants were annotated on the dbSNPvs130 [28], SIFT [29] and Polyphen-2 [30] databases using ANNOVAR software [31]. Next, the Nxtgen2plink.rb software [25] was used to merge individual BAM and PILEUP files that were used for association analysis.

To carry out association analysis, we estimated the minor allele frequency (MAF) of the variants identified in our discovery cohort of 94 Italian idiopathic DVT cases and 98 matched healthy controls. Rare variants were defined as MAF ≤ 1% and common and low-frequency variants as those with MAF > 1%. The association of these coding variants with DVT was performed by individual-variant testing using two-sided Fisher’s exact test and by calculating odds ratio (OR) and 95% confidence interval (CI). In addition, we performed functional annotation of each variant identifying synonymous, non-synonymous or missense variants predicted to be damaging by SIFT [29] and Polyphen-2 [30] variant predictor databases. Genetic association testing was carried out using Plink v1.07 [32] and R programming language [33].

### Replication

The Italian replication cohort consisted of 585 idiopathic DVT cases and 550 matched healthy controls. The top association of the rs2232710 variant with DVT was further tested in two Dutch studies: in 454 DVT cases and 451 controls of the Leiden Thrombophilia Study (LETS) [34] and in 3799 cases with a DVT and/or pulmonary embolism and 4399 controls of the Multiple Environmental and Genetic Assessment of Risk Factors for Venous Thrombosis Study (MEGA) [35]. Replication was performed using TaqMan genotyping at the Leiden University Medical Center, Leiden, Netherlands. The obtained results were subjected to chi-squared statistical test. To assess the association of all variants with DVT, OR and 95% CI were calculated. The *P* values were two-sided and *P*<0.05 were considered statistically significant. Bonferroni correction was used to correct for multiple testing. Genetic association testing was carried out using Plink [32] and R programming language [33]. Quantile-Quantile (QQ) plots of p-value distributions for common and low-frequency (MAF > 1%) and rare variants (MAF ≤ 1%) were generated using R programming language [33].

## Results

To assess the potential role of coding variants underlying genetic predisposition to DVT, we applied targeted next-generation DNA sequencing of the coding area of 186 hemostatic and proinflammatory genes in 94 idiopathic DVT patients and 98 matched healthy controls. As previously described by Lotta et al. [26], quality control and variant filtering criteria such as consensus in the presence of mismatches, read-quality and base-quality parameters, allele balance and strand bias were used to distinguish genetic variants from sequencing errors. In this way, we identified 4366 SNVs and 187 indels in the 192 individuals that were sequenced with an average read-depth of 44 over each site. To assess the risk of DVT in individuals carrying these variants, we calculated minor allele frequencies in cases and controls and performed single variant association analysis using two-sided Fisher’s exact test. In this manner, we obtained a list of 18 genetic variants in 12 genes that showed marked differences in MAF between cases and controls, and therefore, were selected for further analysis (Table 1).

**Table 1:** Single nucleotide variants identified in targeted NGS pilot study in 94 DVT idiopathic patients and 98 healthy controls.

Gene	Chr.	Genomic position	dbSNP ID	Variant type	Minor allele	Protein change	SIFT	Poly-Phen2	Cases		Controls		OR (95% CI)	*P*-value
									Alleles	MAF	Alleles	MAF		
**ZPI/ SERPINA10**	14	94750486	rs2232710	missense	C	Q384R	Dam	Ben	5	0.03	0	0	NC	0.03
**HTR3B**	11	113803028	rs1176744	missense	C	Y129S	Ben	Ben	47	0.25	67	0.34	0.64 (0.4–1)	0.17
**HTR3B**	11	113803666	rs17116138	missense	A	V183I	Ben	Ben	1	0.01	7	0.04	0.14 (0.02–1.2)	0.07
**HIF1A**	14	62207557	rs11549465	missense	T	P582S	Ben	Ben	23	0.12	11	0.06	2.3 (1.1–5)	0.05
**ADRB2**	5	148206440	rs1042713	missense	A	G16R	Ben	Ben	70	0.37	53	0.27	1.6 (1.04–2.5)	0.15
**F13A1**	6	6196141	rs5978	intron	T	-	-	-	37	0.20	20	0.10	2.2 (1.2–3.8)	0.03
**F13A1**	6	6174856	rs5986	syn.	G	E/E	-	-	30	0.16	14	0.07	2.5 (1.3–4.8)	0.02
**TMEM116**	12	112378768	rs7133881	intron	C	-	-	-	4	0.02	0	0	NC	0.06
**PRKCA**	17	64685078	rs2227857	syn.	A	L/L	-	-	55	0.29	85	0.43	0.54 (0.4–0.8)	0.06
**COL4A1**	13	110839550	rs536174	missense	A	T555P	Ben	Ben	17	0.09	32	0.16	0.51 (0.3–0.95)	0.07
**COL4A2**	13	111111235	rs7990383	missense	A	R517K	Ben	Ben	77	0.41	60	0.30	1.6 (1.03–2.4)	0.16
**COL4A2**	13	111119396	rs3803230	missense	C	G683A	Ben	Ben	19	0.10	38	0.19	0.47 (0.26–0.8)	0.03
**COL4A2**	13	111098226	rs4103	syn.	T	P/P	-	-	77	0.41	109	0.55	0.55 (0.4–0.83)	0.11
**COL4A2**	13	111109670	rs9515217	intron	C	-	-	-	9	0.05	34	0.17	0.24 (0.1–0.51)	0.001
**COL4A3**	2	228111435	rs10178458	missense	C	L141P	Ben	Ben	32	0.17	52	0.26	0.6 (0.4–0.93)	0.1
**COL6A2**	21	47542779	rs17357592	intron	T	-	-	-	53	0.28	27	0.14	2.5 (1.5–4.1)	0.01
**COL6A2**	21	47541986	rs9976026	intron	C	-	-	-	49	0.26	22	0.11	2.8 (1.6–4.8)	0.002
**COL6A3**	2	238262021	rs36117715	missense	T	P2218L	Dam	Ben	11	0.06	2	0.01	6 (1.3–28)	0.02

Chr. Chromosome; REF—reference allele sequence; ALT—alternative allele sequence; MAF—minor allele frequency; OR—odds ratio; 95% CI– 95% confidence interval. SIFT and PolyPhen2 predictions: Syn.–synonymous variant; Dam—damaging, predicted to affect protein function; Ben—benign (tolerated), predicted to have no effect on protein function; NC—not calculable.

Among these 18 genetic variants, we identified nine SNVs in genes encoding alpha chains 1, 2 and 3 of Collagen Type IV (*COL4A1*, *COL4A2*, *COL4A3* genes) and alpha chains 2 and 3 of Collagen Type VI (*COL6A2* and *COL6A3*). Furthermore, we found two missense variants rs1176744 and rs17116138 in the 5-Hydroxytryptamine (Serotonin) Receptor 3B (*HTR3B*) gene and an intron variant rs5978 and a synonymous variant rs5986 in the Coagulation Factor XIII, A1 Polypeptide (*F13A1*) gene. Three genes *ZPI*, *HIF1A* and *ADRB2* carried missense substitutions and the *TMEM116* and *PRKCA* genes carried intron and synonymous variants, respectively (Table 1).

The majority of SNVs identified were common or low-frequency variants with the minor allele frequencies above 1% (Table 1). In addition, we identified three rare variants, of which two, the rs2232710 missense variant in the *ZPI* gene and the rs7133881 intron variant in the *TMEM116* gene were particularly interesting as we did not detect any risk allele in the controls, indicating that they are probably rare or low-frequency in the general population. The remaining rare variant was the rs36117715 missense substitution in the *COL6A3* gene. Furthermore, we identified 10 variants with the risk allele frequencies higher in patients than in the healthy individuals, indicating that they could be risk factors for DVT. The remaining SNVs showed the opposite trend, suggesting a protective role in the development of this disorder (Table 1).

Ten of these 18 variants were non-synonymous missense mutations that resulted in amino acid substitutions (Table 1). The remaining eight variants were either intronic or synonymous changes. To assess the putative impact of an amino acid substitution on the structure and function of the protein, we performed an *in silico* analysis of all ten missense variants using SIFT and PolyPhen-2 variant predictor databases. We identified two SNVs, rs2232710 in the *ZPI* gene and rs36117715 in the *COL6A3* gene that were predicted to be damaging by SIFT prediction (Table 1). The rs2232710 SNV encodes a missense change of Glutamine to Arginine at position p.Gln384Arg (Q384R) that has been previously shown to impair the inhibitory activity of the ZPI protein towards activated factor X (FXa) *in vitro* [20]. The rs36117715 SNV encodes Proline to Lysine amino acid change at position p.Pro2218Lys (P2218L) and its effect on the structure or function of the Col6a3 protein is unknown.

**Fig 1:**
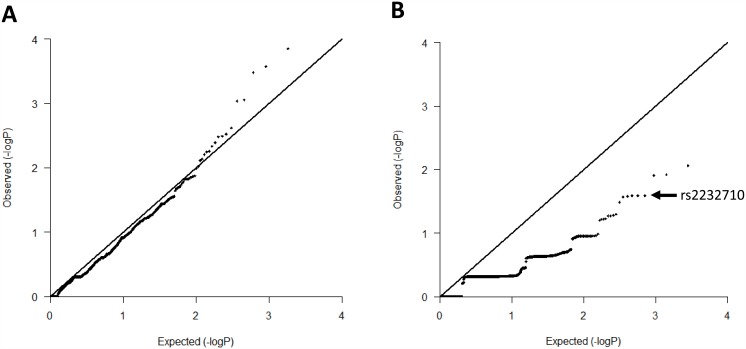
Quantile-Quantile (QQ) plot of p-value distributions for A. common and low-frequency variants (MAF > 1%) and B. rare variants (MAF ≤ 1%). An arrow indicates the rs2232710 variant.

Although the advances in NGS technologies have significantly improved sequencing fidelity, the NGS data is still frequently plagued with false-positives and false-negatives [36], which often leads to signal inflation. For this reason, we generated QQ plots of p-value distributions of common and low-frequency (MAF > 1%) and rare variants (MAF ≤ 1%). As shown in Fig 1, the distribution of p-values of common and low-frequency variants showed good agreement with the expected distribution, suggesting low bias due to population stratification or technical artifacts. Rare variants however, showed a deflated p-value distribution. Since a variant can be a true or false positive in context of both inflated and deflated QQ plot, we decided to replicate all 18 variants identified by targeted sequencing. We applied TaqMan genotyping replication strategy in 585 Italian idiopathic DVT cases and 550 healthy controls. The number of DVT cases and healthy controls carrying a given risk allele in respect to an effective sample size as well as minor allele frequencies in both cases and controls are reported in Table 2. The only variant associated with DVT in the DVT-Milan replication cohort was the rs2232710 missense substitution in the *ZPI* gene with OR = 2.74 (95% CI 1.3–5.7; *P* = 0.0045; Bonferroni *P* = 0.081). We observed the rs2232710-C risk allele in 29 Italian idiopathic DVT cases and 10 healthy controls, corresponding to a MAF of 0.025 and 0.009 for cases and controls, respectively. For the other 17 SNVs, the risk alleles were equally distributed between cases and controls (Table 2), suggesting that these are not susceptibility loci for DVT. The discrepancy between the MAF obtained from our targeted sequencing and replication of the rs536174 SNV in the *COL4A1* gene is most likely an NGS error. The raw data from our Italian replication population is presented in S1 Table.

**Table 2:** Association results of the 18 variants identified in the NGS and replicated in up to 585 idiopathic DVT patients and 550 healthy controls from the DVT-Milan study. Analysis was performed using chi-squared test.

Gene	Chr.	dbSNP ID	Cases		Controls		Effective sample size	OR (95% CI)	*P*-value	Bonferroni *P*-value
			Alleles	MAF	Alleles	MAF				
**ZPI/ SERPINA10**	14	rs2232710	29	0.025	10	0.009	1119	2.74 (1.3–5.7)	0.0045	0.081
**HTR3B**	11	rs1176744	317	0.324	311	0.343	1118	0.9 (0.8–1.1)	0.34	1
**HTR3B**	11	rs17116138	24	0.021	26	0.024	1119	0.9 (0.5–1.5)	0.58	1
**HIF1A**	14	rs11549465	143	0.134	132	0.132	1118	1 (0.8–1.3)	0.88	1
**ADRB2**	5	rs1042713	339	0.374	321	0.365	1113	1 (0.9–1.2)	0.65	1
**F13A1**	6	rs5978	293	0.301	274	0.300	1117	1 (0.8–1.2)	0.98	1
**F13A1**	6	rs5986	125	0.116	117	0.116	1118	1 (0.8–1.3)	0.96	1
**TMEM116**	12	rs7133881	162	0.154	164	0.163	1112	0.9 (0.7–1.2)	0.56	1
**PRKCA**	17	rs2227857	322	0.338	314	0.352	1116	0.9 (0.8–1.1)	0.48	1
**COL4A1**	13	rs536174	0	0	1	0.002	1104	NC	0.14	1
**COL4A2**	13	rs7990383	347	0.389	326	0.373	1118	1.1 (0.9–1.3)	0.44	1
**COL4A2**	13	rs3803230	153	0.141	156	0.153	1119	0.9 (0.7–1.2)	0.43	1
**COL4A2**	13	rs4103	453	0.522	416	0.521	1113	1 (0.9–1.2)	0.96	1
**COL4A2**	13	rs9515217	121	0.108	128	0.122	1118	0.9 (0.7–1.1)	0.3	1
**COL4A3**	2	rs10178458	208	0.203	206	0.207	1111	1 (0.8–1.2)	0.83	1
**COL6A2**	21	rs17357592	166	0.154	171	0.169	1044	0.9 (0.7–1.1)	0.21	1
**COL6A2**	21	rs9976026	159	0.177	171	0.194	1015	0.9 (0.7–1.1)	0.32	1
**COL6A3**	2	rs36117715	65	0.058	49	0.046	1117	1.3 (0.9–1.9)	0.2	1

Chr. Chromosome; MAF—minor allele frequency; OR—odds ratio; 95% CI– 95% confidence interval; NC—not calculable.

To confirm the association of the rs2232710 missense variant with DVT, we decided to replicate this variant in two independent Dutch studies: in 454 cases and 451 controls in the LETS study [34] and in 3799 cases and 4399 controls in the MEGA study [35]. Neither of the Dutch cohorts confirmed our previously obtained association with an OR = 0.71 (95% CI 0.36–1.39) and OR = 0.92 (95% CI 0.74–1.16) for LETS and MEGA, respectively (Table 3). Indeed, we observed the rs2232710-C risk allele equally distributed between cases and controls in both LETs (controls 0.023, cases 0.016) and MEGA (controls 0.019, cases 0.018) (Table 3).

**Table 3:** Replication analysis of the rs2232710 (*SERPINA10/ZPI* gene) single nucleotide variant in patients with idiopathic deep vein thrombosis of lower extremities and heathy controls belonging to three independent cohorts: DVT-Milan, LETS and MEGA. Analysis was performed using chi-squared test.

	rs2232710 genotype					
	TT	TC	CC	MAF	OR (95% CI)	*P* value
**DVT-Milan**						
Controls	529	10	0	0.009	1 (ref)	
Cases	551	29	0	0.025	2.74 (1.3–5.7)	0.0045
**LETS**						
Controls	451	19	1	0.023	1 (ref)	
Cases	454	13	1	0.016	0.7 (0.4–1.4)	0.32
**MEGA**						
Controls	4399	144	14	0.019	1 (ref)	
Cases	3799	127	5	0.018	0.9 (0.7–1.2)	0.48
**TOTAL**						
Controls	5379	173	15	0.018	1 (ref)	
Cases	4804	169	6	0.018	1 (0.8–1.2)	0.98

DVT-Milan—Deep Vein Thrombosis Milan Study; LETS—Leiden Thrombophilia Study; MEGA—Multiple Environmental and Genetic Assessment of Risk Factors for Venous Thrombosis Study; TT—reference genotype; TC—heterozygote for rs2232710-C risk allele; CC—homozygote for rs2232710-C risk allele; MAF—minor allele frequency; OR—odds ratio; CI—confidence interval.

## Discussion

In this manuscript, we report the results of targeted next-generation DNA sequencing of the coding area of 186 hemostatic and proinflammatory genes in 94 idiopathic DVT cases and 98 matched healthy controls employed to assess the potential role of coding variants underlying genetic predisposition to DVT. Using this approach we identified 18 single nucleotide variants in 12 genes with putative role in DVT. Replication of these variants in 585 Italian idiopathic DVT cases and 550 healthy controls from the DVT-Milan cohort revealed the rs2232710 variant in the *ZPI* gene as putative risk factor for DVT. However, independent replication in the two Dutch studies LETS and MEGA did not reproduce this finding. These results confirmed in a large replication cohort the lack of association of the rs2232710 variant with DVT that was previously observed by Young et al. [20].

The reasons for the different prevalence of the rs2232710-C risk allele among controls and cases in DVT-Milan replication population and in the two independent Dutch cohorts LETS and MEGA as well as that of Young et al. [20] may be the different recruitment criteria for patients and controls, different ancestral origin or a random effect due to the relatively small Italian replication cohort. Since other genetic risk factors such as FVL and prothrombin G20120A variants or deficiencies in natural anticoagulants (protein C, protein S and antithrombin) can influence the predisposition to develop venous thrombosis, patients carrying these defects were excluded from our analysis. In the study conducted by Young et al. [20], only the presence of the FVL variant was reported leaving all the other thrombophilia markers unchecked. The different patient recruitment criteria may also be a reason for the negative replication of the rs2232710 in the Dutch population where patients with provoked and unprovoked first events of deep vein thrombosis or pulmonary embolism have been included [34,35].

Another explanation might be the different ancestral origin of the replication cohorts. Our discovery and first replication cohorts consisted mainly of the Southern European Italian ancestry, whereas, that of LETS and MEGA were mainly of the Dutch origin and that of Young et al. [20] of the white British ancestry of New Zealand. Indeed, comparing our study with that of Young et al. [20] we noticed that with the similar size of the replication cohorts, we observed marked differences in the prevalence of the rs2232710-C risk allele in DVT patients compared to controls (5% and 1.9%, respectively), while the previous study did not. We also found differences in MAF in controls coming from our DVT-Milan study and those from LETS and MEGA studies. In comparison to the frequency of the rs2232710-C risk allele in controls from the DVT-Milan, we observed a 2.5-fold and 2.1-fold increase in MAF of controls from LETS and MEGA, respectively. Moreover, we found the rs2232710-C risk allele present in homozygous state (rs2232710-C/C) in both cases and controls in the LETS and MEGA studies but none in our DVT-Milan study (Table 3). To verify this result, we analyzed the prevalence of the rs2232710-C risk allele in healthy European individuals in the 1000 Genomes project phase 3 [37] and the HapMap project phase 3 [38]. We found that in the 1000 Genomes project, the MAF for Italian (Population of Tuscany; TSI) and Iberian populations were identical (0.9%) to that obtained from our replication in the heathy Italian individuals. In comparison, the allele frequency found in the British population from England and Scotland was 1.6%. Similar estimates were obtained when looking at the HapMap project with the MAF of 1.1% and 1.8% for the Italian Tuscany population and general European population, respectively. Since the results of both the 1000 Genomes and the HapMap projects were based on relatively small number of alleles sequenced, we decided to check the estimates for the rs2232710-C risk allele in the Exome Variant Server (URL: http://evs.gs.washington.edu/EVS/; accessed on 18 September 2015) and we found the minor allele frequency for our variant to be 1.1% (with sequences from 8502 alleles from 4251 European Americans). Based on these observations, especially when considering that the healthy Italian and the Iberian individuals harbor similar allele frequencies, one is tempted to speculate about a potential prothrombotic role of the rs2232710-C risk allele solely in the Southern European populations. However, a large-scale genotyping studies in the Southern European ancestries would have to be conducted to confirm the rs2232710 variant as a risk factor for DVT in the Southern European populations.

Similar, however opposite effects have been observed for a nonsense mutation, Trp303Stop (W303X), in the *ZPI* gene. The W303X has been identified as risk factor for DVT in the New Zealand white British ancestry [10]. In the Southern European Spanish and Italian ancestries however, the presence of the W303X mutation is rare and is not associated with DVT [11–13], suggesting that it may have a thrombotic relevance only in the Northern European ancestries. However, sampling error due to the limited replication cohorts may have influenced the results obtained for the rs2232710 SNV in our Italian population as well as those obtained by Van der Water et al. [10] for the W303X mutation. In fact, correcting the results of our Italian replication analysis for multiple testing using the Bonferroni correction revealed borderline significance for the rs2232710 variant (Table 2). In addition, QQ plot analysis of the p-value distribution of rare variants showed a greatly deflated signal (Fig 1). Although this kind of behavior is frequently observed in p-value distributions of rare variants where large *P* values are obtained due to the rarity of their allele counts, it may also reflect bias based on population stratification, differences between cases and controls, lack of statistical power or sequencing errors.

In conclusion, our NGS analysis revealed 18 variants with putative role in DVT. One of them, a missense variant rs2232710 (Q384R) in the *ZPI* gene, was associated with DVT in the Southern European, Italian replication cohort. An independent, large-scale replication in two Dutch cohorts did not confirm this association, implying that the presence of this variant does not increase the risk for venous thrombosis.

## Supporting Information

S1 Table The raw data from our Italian replication population. (XLSX)
